# Anxiety: An overlooked confounder in the characterisation of chronic stress-related conditions?

**DOI:** 10.1371/journal.pone.0230053

**Published:** 2020-04-16

**Authors:** Monet Viljoen, Rohan M. Benecke, Lindi Martin, Rozanne C. M. Adams, Soraya Seedat, Carine Smith

**Affiliations:** 1 Department of Physiological Sciences, Science Faculty, Stellenbosch University, Stellenbosch, South Africa; 2 Department of Psychiatry, Faculty of Health Sciences, Stellenbosch University, Bellville, Cape Town, South Africa; 3 Flow Cytometry and Imaging Unit, Central Analytical Facility, Stellenbosch University, Stellenbosch, South Africa; Harvard Medical School, UNITED STATES

## Abstract

Although anxiety disorders are among the most prevalent of psychiatric disorders, childhood trauma-related studies seldom consider anxiety proneness as distinct aetiological contributor. We aimed to distinguish between trauma- and anxiety-associated physiological profiles. South African adolescent volunteers were categorised for trauma exposure (CTQ, mean score 39±11) and anxiety proneness (AP)(CASI, mean score 37±7, STAI-T, mean score 41±8). Circulating hormone and leukocyte glucocorticoid receptor levels, as well as leukocyte functional capacity, were assessed. AP was associated with lower DHEAs (P<0.05) and higher leukocyte GR expression (P<0.05). DHEAs was also negatively correlated with anxiety sensitivity (CASI, P<0.05). In conclusion, AP may have more predictive power than trauma in terms of health profile. Increased glucocorticoid sensitivity previously reported after trauma, may be a unique function of anxiety and not trauma exposure *per se*. DHEAs concentration was identified as potentially useful marker for monitoring progressive changes in HPA-axis sensitivity and correlated with psychological measures of anxiety.

## Introduction

The advent of modernity raised the general quality of life. However, there are still many people who live in high risk environments, especially in developing countries such as South Africa. Lower socioeconomic standing increases the likelihood of experiencing negative life events and children and young adults in particular are at risk of experiencing early life trauma and poorer health outcomes [1,2]. [3,4] Similarly, behavioural and emotional problems are now common in young children globally [5]. The onset of an anxiety disorder is typically in childhood or early adolescence [6]. In this context, a history of childhood maltreatment has been consistently linked to an increased risk for development of an anxiety disorder [7,8]. Furthermore, anxiety disorders have also been associated with anxiety proneness [9], a function of anxiety sensitivity and trait anxiety [10], which has also been implicated as risk factor for poor performance in neuropsychological domains [11]. From the trauma literature, it is clear that there is high prevalence of anxiety developing as an outcome to trauma exposure. Therefore, it is easy to view anxiety as a symptom of trauma exposure and to overlook the role that pre-existing anxiety may play in health outcome independently of the trauma.

We believe that a relative inconsideration for anxiety as confounder, may explain at least in part, the disparity in physiological maladaptation reported to result from trauma exposure. A representative summary of literature illustrating this disparity is presented in Table 1. Although there is also variability between studies in terms of the general cohort characteristics, the disease context, and the subtype of trauma involved, the inconsistency (and sometimes even contradictory nature) of effects reported is in line with an interpretation of anxiety as potential major confounder of health outcome. Of direct relevance to the current study, systemic maladaptive effects of trauma are commonly ascribed to the dysfunction of the hypothalamic-pituitary-adrenal (HPA) -axis [12]. However, similarly, studies reporting on (non-trauma) anxiety-linked maladaptation also implicate HPA-axis dysfunction (most commonly in terms of cortisol or glucocorticoid receptor levels) as central maladaptive mechanism [13,14]. Furthermore, in terms of the pro-inflammatory phenotype shift that is linked to HPA-axis dysregulation, upregulation of inflammatory markers such as interleukin -6 (IL-6) has been reported in the context of trauma [15,16,17], with similar findings in anxiety related research [18,19]. Taken together, the contradictory data on trauma-associated health outcome and the similarities between trauma and anxiety in terms of reported pro- and anti-inflammatory signalling, suggests that anxiety–in isolation or as component of trauma–may be a more important driver of the physiological changes commonly ascribed to trauma, than trauma itself.

**Table 1:** Representative summary of literature illustrating contradictory results reported for anxiety and trauma in the context of physiological dysregulation.

Title	Authors	Cohort	Diagnosis	Main finding	Outcome
Childhood Maltreatment And Response To Cognitive Behavioural Therapy (CBT) Among Individuals With Social Anxiety Disorder	Bruce, Heimberg, Goldin, & Gross, 2013 [20]	68 treatment seeking out-patients	Generalized Social Anxiety Disorder (SAD)	No form of childhood maltreatment moderated response to CBT for SAD	no CTQ association with anxiety
Childhood Maltreatment Linked To Greater Symptom Severity And Poorer Quality Of Life And Function In Social Anxiety Disorder	Simon et al., 2009 [21]	103 individuals with GSAD (n = 72 male)	Participants who met DSM-IV criteria for GSAD	Emotional abuse and neglect are associated with greater anxiety symptom severity as well as deceased resilience and quality of life	Specific CTQ criteria correlated with anxiety
Childhood Life Events And Childhood Trauma In Adult Patients With Depressive, Anxiety And Comorbid Disorders Vs. Controls	Hovens et al., 2010 [22]	n = 1931 Netherlands Study of Depression and Anxiety (NESDA)	Major depressive disorder, dysthymia, panic disorder with or without agoraphobia, social phobia and generalzed anxiety disorder	Emotional neglect, psychological abuse, physical abuse and sexual abuse were correlated with increased likelyhood of anxiety and depressive disorders	Specific CTQ criteria associated with anxiety, more pronounced in terms of depressive symptoms as well as in comorbid group
Glucocorticoid Receptor Gene Methylation Moderates The Association Of Childhood Trauma And Cortisol Stress Reactivity	Alexander et al., 2018	200 Caucasians (n = 100 female)	No psychiatric diagnosis, history of childhood trauma	DNA methylation of NR3C1 predicts HPA dysregulation in moderate to severe childhood trauma exposure	CTQ associated with stress pathway
Impact Of Physical Or Sexual Childhood Abuse On Plasma DHEA, DHEA-S And Cortisol In A Low-Dose Dexamethasone Suppression Test And On Cardiovascular Risk Parameters In Adult Patients With Major Depression Or Anxiety Disorders	Kellner et al., 2018 [23]	42 childhood trauma patients, 50 controls (n = 92)	Major depressive disorder or anxiety disorder, excluding current diagnosis of psychosis or substance-related disorder	Increase in DHEA levels could not be replicated, however predexamethasone levels were decreased with CT. Physical and sexual abuse were associated with increased TNF-α and IL-6 levels	Specific CTQ criteria linked to increase in stress biomarkers
Childhood Emotional Neglect And Oxytocin Receptor Variants: Association With Limbic Brain Volumes	Womersley et al., 2019	63 Caucasian (n = 35 female)	Social anxiety disorder	Oxcytosin receptor polymorphism rs2254298 shows reduced hippocampal and amygdalar volume	Specific CTQ subtype linked to changes in anxiety-related brain regions
The Impact Of Early Life Stress On Anxiety Symptoms In Late Adulthood	Lähdepuro et al., 2019	n = 1872 (n = 1082 male)	Participants completed anxiety questionnaire (Beck Anxiety Inventory)	Emotional and physical childhood trauma as well as low socioeconomic status was associated with higher anxiety levels in adulthood	Cumilative CTQ linked to increased anxiety symptoms in adulthood
Social Anxiety Disorder And Childhood Trauma In The Context Of Anxiety (Behavioural Inhibition), Impulsivity (Behavioural Activation) And Quality Of Life	Bruijnen, Young, Marx, & Seedat, 2019	n = 102 total particicpants, n = 51 SAD and CT, n = 25 SAD, n = 26 age and gender matched controls	Social anxiety disorder	SAD symptom severity was correlated to childhood trauma exposure	
Childhood Abuse Predicts Affective Symptoms Via HPA Reactivity During Mother-Infant Stress	Kern & Laurent, 2019	47 Females	Participants had no psychosis and CTQ measurement was taken	CTQ correlated with increased cortisol reactivity	Increased CTQ linked to increase in anxiety pathway activity
Anxiety Disorders In Childhood Are Associated With Youth IL-6 Levels: A Mediation Study Including Metabolic Stress And Childhood Traumatic Events	de Baumont et al., 2019	n = 73, n = 41 cases, n = 32 controls	Generalized anxiety disorder, social anxiety disorder, and seperation anxiety disorder	Anxiety disorder diagnosis was associated with higher levels of IL-6 and lower levels of BDNF. HDL cholesterol mediated IL-6 findings	Youth anxiety predicts adulthood stress hormone increase
Impact Of Childhood Life Events And Trauma On The Course Of Depressive And Anxiety Disorders	Hovens et al., 2012 [24]	n = 1209 Netherlands Study of Depression and Anxiety (NESDA)	Major depressive disorder and Dysthymia. Panic disorder, Agoraphobia, Social Phobia and Generalized Anxiety Disorder	Emotional neglect, psychological and physical abuse predict comorbidity of depressive and anxiety disorders. However, childhood trauma had a larger effect on depressive outcomes	Specific CTQ criteria predicts comorbidity of anxiety and depression

In further support of this argument, in the non-trauma literature, it has long been known that anxiety is implicated in the aetiology of chronic disease. For example, in a previous report of increased prevalence of chronic disease (e.g. diabetes, arthritis, cardiac pathology) in individuals suffering from generalised anxiety disorder, the authors argued that anxiety symptoms should not be discounted as a mere by-product of other “medical illness” [25]. More recently, associations between the prevalence of anxiety and multi-morbidity was reported in older adults [26]. These respected authors cautioned that the causative direction of this association can only be confirmed by prospective studies. From these reports, it is clear that the presence of anxiety–regardless of its cause–impacts significantly on longer term health outcome.

Turning attention to the specific population most at risk of adverse health outcome after trauma, adolescents are at a critical stage where early life experiences are being manifested within a more adult-like neurocognitive paradigm [27]. Furthermore, at adolescent age, a shorter time period has passed from the trauma exposure to assessments made, so that allostatic load is a lesser confounding factor than in adults. Considering these facts, as well as the fact that anxiety-disorders manifest during early adulthood, an adolescent population is the ideal population in whom to study maladaptation *before* clinical disease onset, so that appropriate avenues for intervention may be elucidated.

Childhood maltreatment trauma in particular has been associated with severe and lasting effects on behavioural and emotional functioning [28]. The most drastic effects occur within the cortical and limbic systems predominantly, with the amygdala, hippocampus and prefrontal cortex showing structural alterations that correlate with maltreatment measures [28,29,30]. Although there seems to be sufficient evidence to suggest significant alterations to brain matter density and structure, there are still some gaps within the literature with regard to the specific changes associated with specific subtypes of trauma and how these relate to the physiological changes reported after trauma exposure. This becomes relevant when considering that differential mechanisms are responsible for the volumetric changes associated with trauma [29,31,32]. Furthermore, there may be some overlap between the physiological mechanisms responsible for the above-mentioned changes in the brain and mechanisms that alter inflammatory signalling in the context of chronic stress, where the chronic stress is due to childhood trauma exposure [33,34]. Again, this speaks to the possibility that the driving mechanisms underlying the neuropsychological changes seen in the context of trauma may be intrinsically linked to the specific physiological changes that occur as result of the anxiety paradigm that usually accompanies the experience of trauma.

Therefore, the current study aimed to show the importance of anxiety–which may precede trauma exposure–as causative factor in adaptations of physiological parameters commonly attributed to trauma exposure. Known subtypes of anxiety, as well as childhood trauma exposure severity, was correlated to physiological maladaptation in biological systems in an attempt to point out the relative importance of anxiety in determining health outcome in the context of trauma.

## Materials and methods

### Participant recruitment and ethical considerations

1149 Participants (16–18 years of age) were recruited by systematic random sampling from 31 government schools around Cape Town, South Africa, after obtaining ethical clearance from the Stellenbosch University Human Research Ethics Committee (Reference number: N11/04/131) and permission from the provincial Department of Education. Written informed assent was obtained from all participants, as well as from parents/legal guardians. Research was conducted in accordance to The Declaration of Helsinki.

Recognizing that there are potential confounding factors such as socio-economic status, individual resilience, social and familial support, etc., a particular strength of the current study is that the study cohort was selected from a particularly homogenous demographic, with very similar cultural background, level of formal education, income and ethnicity.

### Psychiatric evaluation and experimental grouping of subjects

All participants were pre-screened for trauma exposure, as well as depression, and alcohol and drug use (the latter three potential confounders were employed as exclusion criteria), using the results of the Centre for Epidemiological Studies Depression Scale for children (CES-DC) [35], Alcohol Use Disorders Identification Test (AUDIT) [36], Drug Use Disorders Identification Test (DUDIT) [37], Adolescent Coping Orientation for Problem Experiences (A-COPE) [38], childhood maltreatment and anxiety-related traits using the Childhood Trauma Questionnaire (CTQ-SF) [39], Child Anxiety Sensitivity Index (CASI) [40] and the trait section of the State-Trait Anxiety Inventory (STAI) [41]. Internal consistency values for these tests have been published elsewhere [42]. Using scores of the CTQ, as well as a composite score of the STAI-T and CASI as a measure of AP, adolescents were classified as follows: a) high childhood maltreatment/trauma and high anxiety-prone (upper 66th percentile for both variables, HI-HI), b) high childhood maltreatment/trauma and low anxiety-prone (upper 66th percentile for childhood maltreatment/trauma and lower 66th percentile for anxiety proneness, HI-LO), c) low childhood maltreatment/trauma and high anxiety-prone (lower 66th percentile for childhood maltreatment/trauma and upper 66th percentile for anxiety proneness, LO-HI) and d) low childhood maltreatment/trauma and low anxiety-prone (lower 66th percentile for both variables, LO-LO). These participants were re-screened by means of a structured diagnostic interview conducted by a qualified research psychologist (LM) to screen for the presence of anxiety and mood disorders using the Mini-International Neuropsychiatric Interview-Kid for children and adolescents (MINI-KID; [43]), which follows the Diagnostic and Statistical Manual of Mental Disorders-IV [44] and WHO International Classification of Diseases-10 (1992) criteria for the diagnosis of psychiatric disorders, screening for 17 Axis I disorders. In addition, confirmation of group status and compliance with inclusion and exclusion criteria were confirmed by administration of the Children’s Depression Inventory (CDI) [45], the Adolescent Drinking Inventory (ADI) [46] and the multidimensional Anxiety Scale for Children (MASC) [47]. Questionnaire scores and psychological assessment outcome measures have been comprehensively reported in association with neuropsychological test results for the larger group [11]. For the purpose of this study, a subgroup of 43 participants were randomly selected (limitation of sample size was required due to high analytical cost). Final size of experimental groups were as follows: HI-HI, n = 9; HI-LO, n = 12; LO-HI, n = 9 and LO-LO, n = 13.

### Blood sample collection and analysis

Early morning fasted blood samples were obtained for isolation of peripheral blood mononuclear cells (PBMCs) and serum analysis for cortisol, prolactin, dehydroepiandrosterone-sulphate (DHEAs) and testosterone levels by automated analysis by automated analysis in pathology laboratory (Pathcare), using standard laboratory procedures.

In addition, whole blood was analysed for leukocyte immunophenotyping and intracellular GR expression using flow cytometry (FASCAria, BD Biosciences, USA). The following monoclonal antibodies were used: FITC-conjugated anti-GR, PE-conjugated anti-CD56 (for NK cells), Alexa Fluor anti-CD19 (for B cells), APC-C7 CD3 (for T cells) and BV421 CD14 (for monocytes) (Biolegend, USA).

In order to properly gate and identify cells, single stain controls and fluorescence minus one (FMO) controls were employed to determine the demarcation of the true positive and negative populations for the markers of interest. For the compensation, Anti-Mouse Ig CompBeads (BD Biosciences, USA) were employed. Cytometer setup and tracking (QC) beads and 8-peak beads were run to ensure instrument linearity. Lastly, an unstained control for each participant was included. For cell staining, the CytoFix/CytoPerm kit was used according to manufacturer’s instructions (BD Biosciences, San Jose, USA).

Cell surface staining was performed by incubating 50 μl of whole blood with 5 μl of each monoclonal antibody in the dark for 30 minutes at room temperature. After incubation, erythrocytes were lysed using BD FACSLyse (BD Biosciences, San Jose, USA), washed with the BD CytoPerm buffer and pelleted at 600xg for 5 minutes. The cells were then re-suspended in BD CytoFix buffer for at 4°C for 20minutes. Thereafter, the cells were washed again, before50μl of CytoPerm—with a predetermined optimal concentration (2·66 μg/ml) of anti-GCR MoAb—was added and cells incubated at 4°C for 20 min in the dark. After washing, to remove any unbound antibodies, the cell pellets were resuspended in 500 μl of 4% paraformaldehyde in PBS and run on the flow cytometer. All analyses were acquired using BD FACSDiva software v6.1.3. A total of 1000 000 events were acquired in the light scatter (FSC/SSC) gate. The fluorescence intensities of GCR-labelled leukocyte populations were displayed and determined as median fluorescent intensity (MFI) values on four-decade histogram plots.

Absolute cell counts were calculated for leukocyte subpopulations that were analysed using flow cytometry. These were derived from a total WBC and lymphocyte count obtained by automated hematology analysis (Celldyne 3700CS), as well as the respective percentages of cells of the lymphocyte gates obtained during flow cytometry analysis. Furthermore, as a quality control (QC) step, the calculated absolute counts of subpopulations were also added together and did sum to the total lymphocyte count.

### Peripheral blood mononuclear cell functional capacity

PBMCs were isolated from whole blood by density gradient centrifugation using Histopaque (Sigma Aldrich, 10771). PBMCs were seeded at 200 000 cells/well in RPMI and stimulated with either media (control) or LPS (Lipopolysaccharide) (50ng/ml) for 6 hours at 37°C. At the end of the 6hr stimulation period, cells were pelleted, supernatant removed and frozen at −80°C. Subsequently batch analysis for concentrations of IL-1β, IL-6, IL-10 and TNF-α (Tumour Necrosis Factor alpha) (detection limits respectively 0.8, 0.9, 1.1 and 0.7 pg/ml) was performed, using Milliplex MAP Human Cytokine Kits (cat# HCYTOMAG-60K, Merck, Darmstadt, Germany) and a Bio-Plex 200 Array Reader (Bio-Rad, Hercules, CA, USA).

### Statistical analysis

Both experimental grouping of subjects and data analysis were performed in consultation with Prof Martin Kidd, an experienced biostatistician (Centre for Statistical Consultation, Stellenbosch University). Depending on normalcy of data distribution, either parametric or non-parametric two-way ANOVA was used to determine main effects, followed by Fisher LSD post hoc testing. For analysis of relationships between parameters, Spearman rank correlation coefficients were calculated. As additional test for robustness, linear regression analyses were performed. Given the fact that multiple comparisons were calculated for complimentary parameters, no correction was required. [48,49] In these analyses gender, anxiety proneness and trauma exposure severity was used as fixed effects, with subject nested in gender*trauma*anxiety as random effect.

## Results

The validity of questionnaires used for experimental grouping of individuals was evident from results obtained, which clearly indicated significantly higher CASI scores in individuals classified as high anxiety prone, while individuals classified as hi trauma exposed scored significantly higher in the CTQ (Table 2). Similarly, our decision to use the composite score for trait anxiety and anxiety sensitivity—anxiety proneness–as anxiety measure in the current study, is validated by the fact that trait anxiety and anxiety sensitivity scores, which were significantly correlated to each other (R = 0.56, P<0.05), were differentially correlated with physiological measures. Anxiety sensitivity was most often correlated with leukocyte counts Pearson coefficient calculation returned correlations between AS and both Eosinophils and Basophils (Eosinophils, Pearson R = -0.3; Basophils, Pearson R = -0.33, P<0.05). Regression analysis supported this and suggested an additional correlation between AS and Monocyte count (Monocytes, F = 6.37, P = 0.016; Eosinophil, F = 4.14, P = 0.048; Basophil, F = 5.092, P = 0.029) In contrast, trait anxiety was more closely correlated with leukocyte GR levels (B Lymphocyte GR, Pearson R = 0.33; Natural Killer Cell GR, Pearson R = 0.36; Monocyte GR, Pearson R = 0.34, P<0.05). Regression analysis supported this finding (B Lymphocyte GR, F = 4.95, P = 0.032; Natural Killer Cell GR, F = 6.23, P = 0.017; Monocyte GR, F = 5.47, P = 0.024) Since glucocorticoid sensitivity is determined by both the GR level on any individual cell and the total number of cells available to present GR for glucocorticoid binding, the composite score of anxiety proneness is arguably the most accurate anxiety-related measure to use in data reduction and interpretation, at least in the current study.

**Table 2:** Accuracy of subject grouping is reflected by significant ANOVA main effects for trauma exposure (CTQ) and anxiety proneness (combined STAI-T and CASI) with subscales of each questionnaire in column 2 and 3.

	HI-HI Mean ± SD	HI-LO Mean ± SD	LO-HI Mean ± SD	LO-LO Mean ± SD	ANOVA effect of trauma exposure F(1,41); P-value	ANOVA effect of anxiety proneness F(1,41); P-value
**STAI-T**	52 ± 7	39 ± 3	46 ± 6	33 ± 6	**F = 5.59; p < 0.01**	**F = 43.96; p < 0.000001**
**CASI total**	42 ± 4	31 ± 4	45 ± 4	33 ± 7	F = 0.61; p = 0.17	**F = 56,66; p < 0.000001**
CASI Social Concerns	12 ± 1	9 ± 2	12 ± 1	10 ± 2	F = 0.06; p = 0.87	**F = 17.47; p < 0.001**
CASI Psychological Concerns	6 ± 1	5 ± 2	7 ± 2	4 ± 1	F = 0.02; p = 0.93	**F = 23.18; p < 0.0001**
CASI Physical Concerns	23 ± 3	17 ± 3	26 ± 2	18 ± 3	F = 1.39, p = 0.24	**F = 47.28; p < 0.000001**
**CTQ total**	50 ± 11	44 ± 9	33 ± 8	31 ± 5	**F = 30.42; p < 0.00001**	F = 1.54; p = 0.22
CTQ Physical neglect	10 ± 3	9 ± 4	8 ± 3	6 ± 1	**F = 7.52; p = 0.01**	F = 1.23; p = 0.27
CTQ Emotional abuse	12 ± 5	9 ± 4	6 ± 2	6 ± 1	**F = 13.09; p < 0.001**	F = 1.73; p = 0.20
CTQ Emotional neglect	13 ± 4	13 ± 5	9 ± 4	8 ± 3	**F = 12.92; p < 0.001**	F = 0.14; p = 0.72
CTQ Physical abuse	9 ± 4	7 ± 3	5 ± 0	5 ± 0	**F = 12.82; p < 0.001**	F = 1.24; p = 0.27
CTQ Sexual abuse	6 ± 2	6 ± 1	5 ± 0	5 ± 1	**F = 4.57; p = 0.03**	F = 0.00; p = 1.00

Statistical data is presented as F-values; P-values. NS = no significant effect

Main findings from current data suggests that physiological outcomes commonly accepted to result from trauma exposure, seemed more consistently associated with anxiety–both state and trait–than with trauma.

### Association of anxiety proneness with glucocorticoid receptor expression

**Fig 1:**
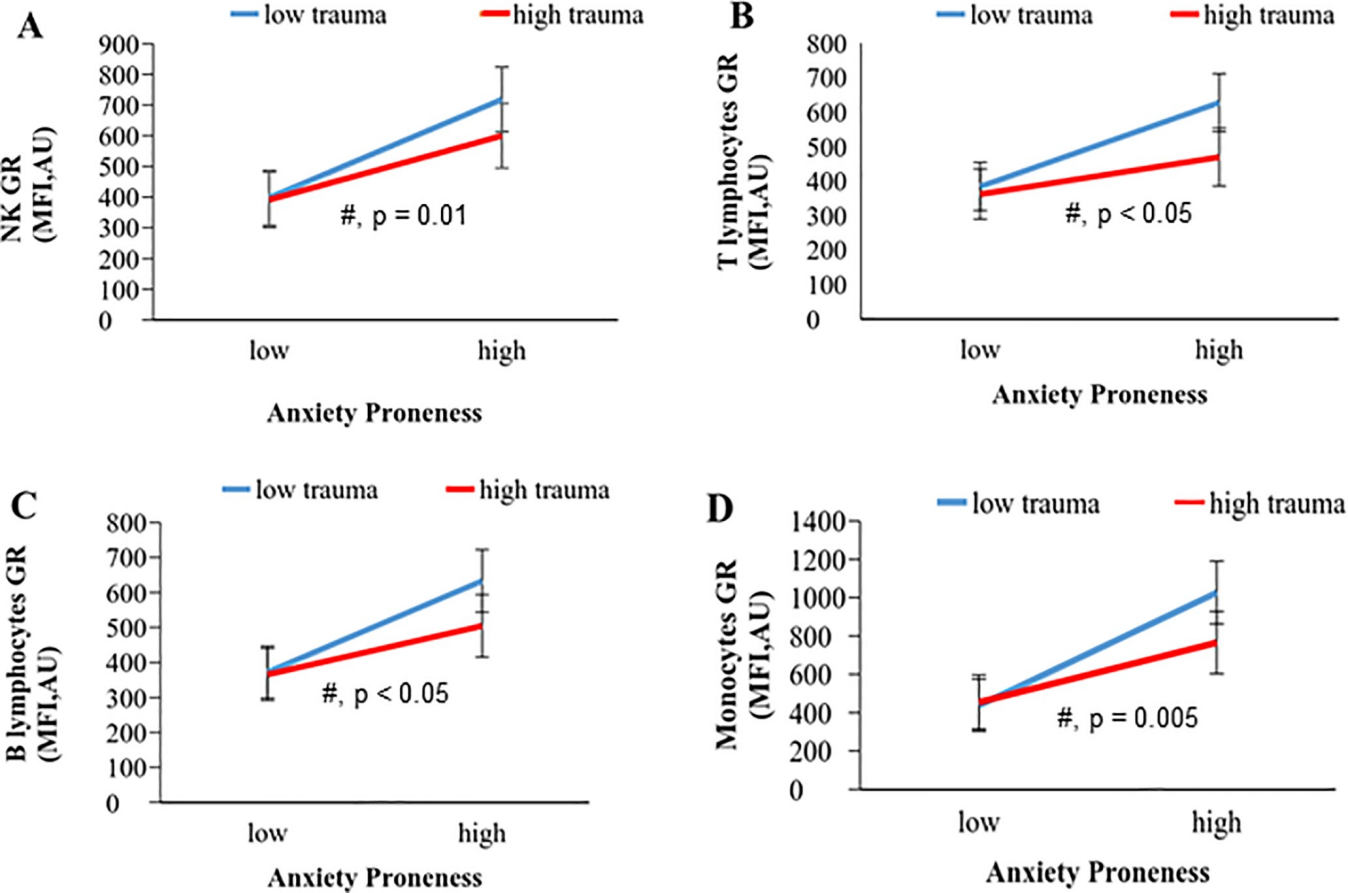
Consistent effect of anxiety proneness on glucocorticoid receptor (GR) expression in leukocyte subpopulations. Statistics: # = ANOVA main effect of AP, Abbreviations: AU, arbitrary units; MFI, mean fluorescent intensity.

A marked increase in expression of GR was seen in AP in all white blood cell (WBC) subpopulations assessed (Fig 1). (Interestingly, when correlating either trait anxiety or anxiety sensitivity to GR levels separately, correlations seen with mainly trait anxiety was less consistent than reported here for AP). Childhood trauma severity (when grouping was done according to total CTQ score) did not influence GR expression statistically. However, it did seem to have an effect on the magnitude of increase associated with anxiety. In the two groups with low AP, values for GR expression were similar. However, in the presence of higher AP, the anxiety-associated increase in GR expression was consistently higher in the low-trauma vs. the high-trauma group (Fig 1), suggesting that trauma exposure may blunt the GR response to anxiety to some extent.

A confirmation of these results—suggestive of somewhat opposing effects of anxiety and trauma—is elucidated when all subjects are grouped together and correlation coefficients was calculated across the continuum for GR expression vs. CTQ, STAI-T and CASI (Table 3). Here, CASI-Physical concerns showed a consistent tendency for a positive correlation with GR expression for all of the cell types assessed, while a consistent negative correlation of CTQ-Physical neglect with B cell (Spearman P = -0.05; R = -0.3), NK cell (P = -0.05; R = -0.29), and T cell (P = -0.03; R = -0.33) GR expression levels became evident.

**Table 3:** Correlations of glucocorticoid receptor expression with trait anxiety, anxiety sensitivity (and subscales) and trauma (and subscales).

Parameter	STAI-T	CASI Social concerns	CASI Psychologi-cal concerns	CASI Physical concerns	CASI total	CTQ Physical neglect	CTQ Emotional abuse	CTQ Emotional neglect	CTQ Physical abuse	CTQ Sexual abuse	CTQ total
B lymph GR: MFI	0.23 (0.135)	-0.01 (0.972)	0.12 (0.443)	0.28 (0.071)	0.22 (0.154)	**-0.30 (0.050)**	0.09 (0.545)	-0.21 (0.171)	-0.14 (0.368)	-0.23 (0.137)	-0.21 (0.173)
NK GR: MFI	0.16 (0.156)	-0.01 (0.948)	0.13 (0.413)	0.29 (0.059)	0.23 (0.131)	-0.29 (0.055)	0.06 (0.069)	-0.23 (0.133)	-0.20 (0.208)	-0.23 (0.141)	-0.25 (0.111)
T Lymph GR: MFI	0.16 (0.306)	-0.04 (0.792)	0.09 (0.550)	0.26 (0.088)	0.20 (0.201)	**-0.33 (0.033)**	0.04 (0.812)	-0.26 (0.096)	-0.16 (0.318)	-0.23 (0.139)	-0.26 (0.090)
Mono GR: MFI	0.22 (0.156)	0.03 (0.857)	0.17 (0.28)	0.26 (0.094)	0.24 (0.122)	-0.19 (0.222)	0.10 (0.512)	-0.22 (0.149)	-0.14 (0.369)	-0.16 (0.297)	-0.18 (0.242)

Data is presented as Spearman r-values (P-values). Significant values and trends are indicated in bold font.

GR = glucocorticoid MFI = mean fluorescent intensity; NK = natural killer cell; T lymph = T lymphocyte; Mono = monocyte

### DHEAs, rather than cortisol, identified as most useful endocrine marker for AP

In terms of serum testosterone levels, there was a main effect of gender, as anticipated, with males exhibiting significantly higher basal testosterone levels than females (21.8 ± 1.3 vs. 2.3 ± 1.0 nmol/L, ANOVA main effect P<0.00001). Testosterone was not further analysed statistically for males, due to low subject number (n = 11). For females, testosterone levels were not related to either trauma or AP.

**Fig 2:**
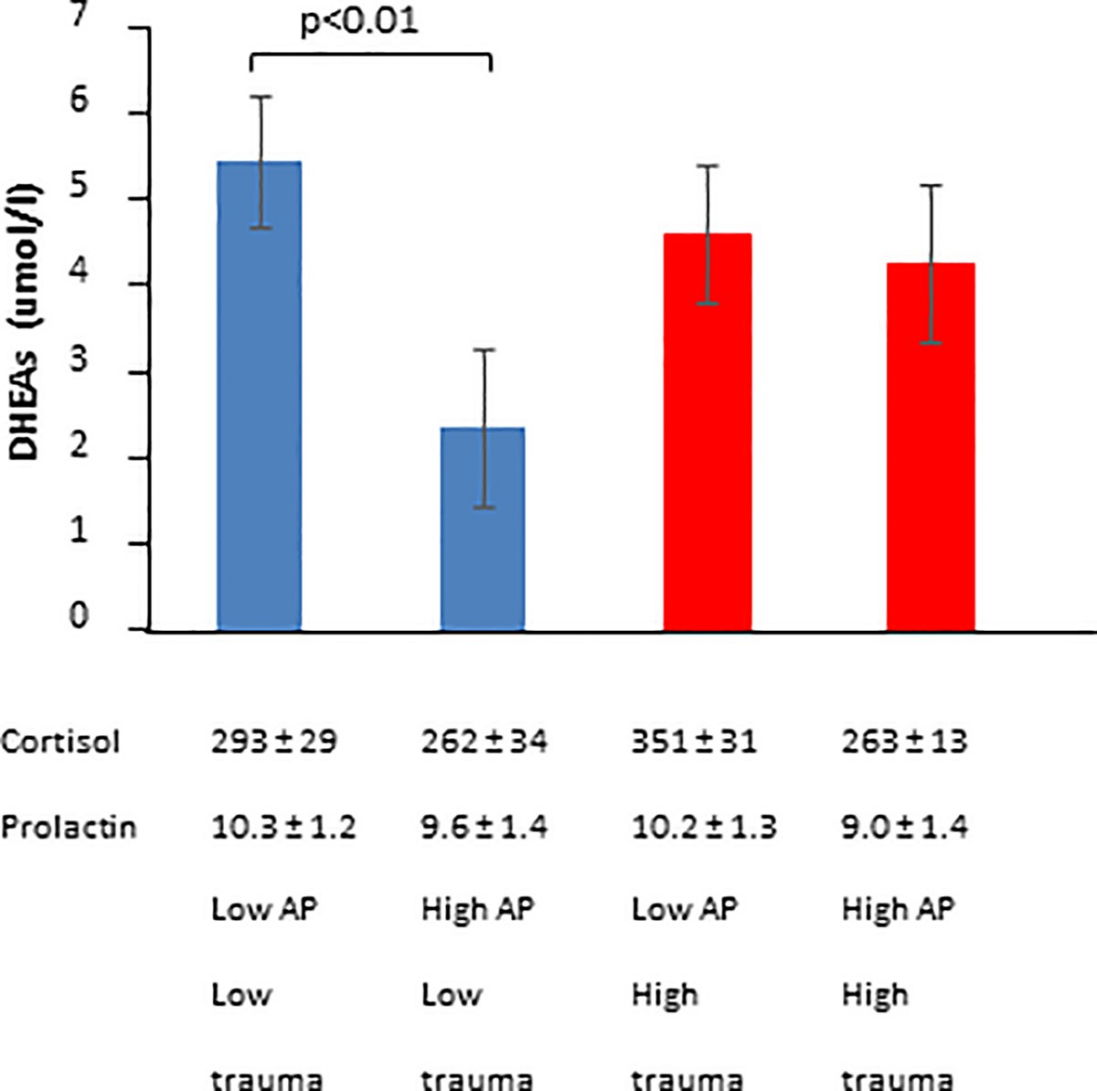
Effect of anxiety proneness and trauma exposure on serum dehydroepiandrosterone-sulfate (DHEAs), cortisol and prolactin levels.

While both serum prolactin and cortisol levels were within normal ranges and not influenced by either trauma or AP, high AP was associated with decreased DHEAs (ANOVA main effect, P<0.05; Fig 2). The latter effect was largely due to a major inhibitory effect in anxiety prone individuals without trauma (Fischer LSD post hoc LO-LO *vs* LO-HI, P = 0.01). Of further importance is the fact that a large portion of this population presented with below-normal DHEAs concentrations: 67% of HI-HI, 75% of HI-LO, 100% of LO-HI and 62% of LO-LO. Also, when correlations across the group as a whole were considered, CASI-Total, CASI-Physical concerns and CASI-Psychological concerns were negatively correlated with basal DHEAs levels (P<0.05, Table 4). Regression analysis confirmed this interpretation (supplementary material).

**Table 4:** Correlations between circulating hormone levels with trait anxiety, anxiety sensitivity (and subscales) and trauma (and subscales).

	STAI-T	CASI Social concerns	CASI Psychologi-cal concerns	CASI Physical concerns	CASI total	CTQ Physical neglect	CTQ Emotional abuse	CTQ Emotional neglect	CTQ Physical abuse	CTQ Sexual abuse	CTQ total
Prolactin (ug/L)	0.09 (0.577)	0.02 (0.892)	0.01 (0.938)	-0.15 (0.356)	-0.07 (0.657)	-0.00 (0.993)	0.12 (0.437)	**0.31 (0.044)**	-0.09 (0.555)	0.21 (0.172)	0.13 (0.405)
DHEAS (umol/L)	-0.22 (0.147)	-0.01 (0.183)	**-0.35 (0.021)**	**-0.37 (0.013)**	**-0.36 (0.017)**	-0.10 (0.532)	0.17 (0.267)	-0.02 (0.902)	0.10 (0.525)	0.03 (0.832)	0.03 (0.852)
Cortisol (nmol/L)	-0.01 (0.954)	0.11 (0.481)	-0.10 (0.514)	-0.10 (0.532)	-0.06 (0.711)	0.03 (0.863)	-0.05 (0.751)	0.12 (0.433)	-0.06 (0.698)	0.24 (0.133)	0.07 (0.665)

Data is presented as Spearman r-values (P-values), Degrees of freedom, n = 41. Significant values and trends are indicated in bold font.

### IL-10 modulation in response to LPS stimulation

In terms of *ex-vivo* cytokine production in response to LPS stimulus no significant effects of either anxiety proneness or trauma history were detected for pro-inflammatory cytokines TNF-α, IL-1β or IL-6, although the relatively high variability of current data (not shown) precludes firm conclusions. In terms of the anti-inflammatory IL-10 specifically, average LPS-induced production levels were highest in the low trauma, low AP group (36.9 ± 8.96 pg/ml), while in comparison, either presence of high trauma (9.7 ± 3.87 pg/ml, P<0.01) or high AP (11.4 ± 6.60 pg/ml, P<0.01) significantly inhibited IL-10 production. The effects of trauma and AP did not appear to be additive (11.3 ± 3.88 pg/ml in high AP, high trauma, P<0.01 when compared to low AP, low trauma).

## Discussion

By simultaneous assessment of physiological and psychological parameters, the current study was able to point out a significant oversight in the literature and identify a potential tool in monitoring the physiological progression of anxiety. Despite the fact that analytical costs limited the subject number somewhat, we are confident that our data analysis was sufficiently stringent and our interpretation of current data sufficiently conservative.

The broader literature clearly supports very different profiles for anxiety and trauma when viewed as separate entities. Although in both conditions variable results for cortisol concentration in circulation have been reported, anxiety generally involves increased CRH and/or GR expression levels and enhanced Dex suppression (denoting enhanced negative central feedback). Interestingly, these physiological features has been reported for both PTSD (e.g. [50]) and melancholia [51], where the construct of anxiety underlies both disorders. In contrast, decreased CRH and/or GR expression levels as well as Dex suppression being overridden (denoting impaired negative central feedback) has been associated with chronic stress (not burn-out) and major or atypical depression (e.g. [52,53]), where anxiety does not necessarily play a major role. Despite these contrasting physiological outcomes reported for anxiety and stress, the confounding effect of anxiety seem to have been largely ignored in studies on trauma exposure–specifically also childhood maltreatment–where the majority of reports fail to even report anxiety levels of individuals. This may explain the contradicting results reported for trauma and the similar effects that have been reported for trauma and anxiety (Table 1). In the current study, an attempt was made to distinguish between anxiety and trauma, and to differentially correlate these measures to physiological outcome parameters. Current data suggest that, in a South African adolescent population with a history of childhood maltreatment, the level of trauma exposure may have a relatively insignificant role to play in central information processing and physiological outcome parameters, when compared to the predictive power of anxiety proneness. This major, novel result highlights the importance of considering anxiety or anxiety proneness as potential confounding factor when assessing effects of childhood trauma.

In terms of endocrine parameters assessed, cortisol has commonly been assessed in the context of stress and allostasis (e.g. as reviewed by [54]), although not in populations presenting with subclinical anxiety, as reported on here. In contrast, dehydroepiandrosterone (DHEA) has only been investigated significantly in models of ageing [55]–where ageing is associated with decreased DHEA concentration—and PTSD, for which literature is growing, but contradictory. In the context of PTSD, a recent study reported increased DHEA levels in war trauma-exposed individuals with PTSD when compared to non-trauma exposed controls [56]. However, in a Serbian study, DHEA levels was reported to be lower in patients with PTSD when compared to non-PTSD trauma-exposed individuals [57]. Interestingly, and relevant to the current discussion, the authors concluded that DHEA failed as trauma indicator, as it was found to be significantly influenced by personality. In terms of adolescent PTSD, both acute (single event rape) and chronic (repeated sexual abuse) trauma was reported to be associated with decreased DHEA concentrations [58,59]. Current data similarly indicates DHEAs levels below the expected normal range for the majority of study participants with high anxiety proneness in the absence of trauma exposure. The low DHEAs may indicate adrenal burnout/allostasis in these individuals, which may point towards an AP-dependent hypoactivity of the lower (excluding the hypothalamus) HPA-axis, which alarmingly is already evident during adolescence. This result is particularly significant, in light of the existing theory of a trajectory from hyper- to hyposensitivity to glucococortioids and the resultant hyper- to hypocortisolism in response to years of childhood maltreatment [60,61]. The fact that DHEA, but not cortisol, showed significant predictive power in the context of AP, is significant. The fact that trauma exposure in the current study, seemed to counter anxiety-related decreases in DHEA, suggests that at least some studies reporting on trauma, may in fact be measuring effects of anxiety, accounting for the contradictory literature. The fact that DHEAs does not have a potentially confounding major role in metabolism, which cortisol does, probably contributes to this result. The consistency of current data implicating DHEAs as sensitive marker in the context of anxiety proneness, warrants further purpose-designed investigation to assess the potential of DHEAs as marker for monitoring anxiety-related progressive changes in HPA-axis sensitivity. In addition, the possibility of using relatively increased DHEAs levels as “warning” indicator in trauma studies to indicate high risk of anxiety as confounding effect, should be investigated.

In terms of the IL-10 result, maladapted cytokine responses have long been implicated in psychological disorders, including depression and anxiety [62,63]. Current results are in agreement with the literature. For example, in a study of over 100 adults [64], anxiety and depression were linked to a relatively more pro-inflammatory profile. Also, chronically stressed mice exhibited decreased hippocampal IL-10 expression [65], while pro-inflammatory cytokine responses were unaltered–as also reported in the current study. Current data is the first to our knowledge, to report IL-10 maladaptation already present in adolescents exposed to childhood maltreatment and/or having high anxiety proneness, in the absence of other apparent causative factors or pathology.

In line with DHEA and IL-10 data, leukocyte glucocorticoid receptor (GR) expression was consistently elevated with AP. This enhanced leukocyte glucocorticoid responsiveness/sensitivity is in line with studies on PTSD (e.g. [66]_40ew0vw) but contrasts with the literature on models of chronic stress, where GR is generally down-regulated [67]. In our study, trauma seemed to mildly blunt the AP-induced increase in GR expression consistently across all leukocyte sub-populations. This may suggest that with chronic stress, GR expression may be down-regulated in response to chronically raised cortisol levels. Hence, increased glucocorticoid sensitivity may be a unique function of anxiety and not trauma exposure. Therefore, we suggest that trauma-focused literature (e.g. related to PTSD and childhood maltreatment) be revisited, to assess whether effects previously ascribed to trauma may in fact be due to AP.

We acknowledge the limitations posed by the relatively small (n = 43) sample size. However, as mentioned, the cohort is robust and homogenous for potential confounders such as socio-economic factors. Nevertheless, to truly dissect the relative contribution of anxiety to health outcome when compared to that resulting from trauma directly, a larger cohort study is preferable.

## Conclusion

Data indicates that in our adolescent population, anxiety proneness exhibited a greater association with physiological profile than childhood maltreatment. Current data further highlight the potential of DHEAs as useful tool for monitoring and/or managing at risk populations in the context of anxiety, but not trauma.

## Supporting information

S1 Analysis (XLSX)

S1 Data (XLSX)
